# Prediction and effect on relapse of natural killer cell alloreactivity based on KIR-HLA interactions in pediatric haploidentical transplantation with anti-thymoglobulin

**DOI:** 10.3389/fimmu.2025.1643244

**Published:** 2025-08-26

**Authors:** Xiang-Feng Tang, Yan-Hui Luo, Ying-Jian Si, Mao-Quan Qin, Wei Lu, Wei Chen, Guo-Sheng Xing, Wei Cao, Hai-Fei Zhou, Xiang-Jun Liu

**Affiliations:** ^1^ National Engineering Laboratory for Birth Defects Prevention and Control of Key Technology, Beijing Key Laboratory of Pediatric Organ Failure, Department of Pediatrics, The Seventh Medical Center of PLA General Hospital, Beijing, China; ^2^ Department of Hematology and Oncology, Beijing Children’s Hospital, Capital Medical University, Beijing, China; ^3^ Beijing BFR Gene Diagnostics, Beijing, China

**Keywords:** allogeneic hematopoietic stem cell transplantation, pediatric haploidentical transplantation, killer immunoglobulin-like receptor, educational model, clinical outcome

## Abstract

**Introduction:**

Relapse continues to be a major factor contributing to therapeutic failure in haploidentical hematopoietic stem cell transplantation (HSCT). The role of natural killer (NK) cell alloreactivity mediated by killer immunoglobulin-like receptors (KIRs) is considered important in postoperative immune reconstitution and in mitigating relapse. However, its clinical implications remain incompletely defined, and its impact on allogeneic HSCT is controversial across studies.

**Methods:**

In the present investigation, we assessed the effect of predicted NK cell alloreactivity through KIR–ligand interactions on relapse and survival outcomes in a pediatric cohort. This retrospective study included pediatric patients who underwent their first haploidentical HSCT following the Beijing protocol between 2013 and 2023. Both low- and high-resolution typing methods were employed for all donor and patient samples. The presence of NK cell alloreactivity was determined using predictive models incorporating the HLA class I molecules of both donors and recipients. NK cell alloreactivity was classified as ALLO or Non-ALLO based on the presence or absence of predicted alloreactivity, and its effects on relapse and overall survival were evaluated through individual and combinatorial interactions.

**Results:**

Multivariate analysis demonstrated that, among patients lacking A3/A11, those who received grafts from donors with both KIR3DL2+ and A3/A11+ had an 86% lower risk of relapse (adjusted hazard ratio 0.136; p = 0.0489). Both Synthesis-iKIR and combined Synthesis-iKIR/KIR2DS1 showed significant independent effects on overall survival, with clinically adjusted hazard ratios of 0.305 and 0.316 (p < 0.005), respectively. The disease state at transplantation was an independent clinical factor influencing prognosis. Other additive models failed to effectively predict clinical outcomes in pediatric recipients.

**Discussion:**

These results indicate that pediatric patients exhibiting NK cell alloreactivity, as predicted by the KIR3DL2–A3/A11 combination, had a significantly lower cumulative incidence of relapse. Furthermore, alloreactivity predicted by Synthesis-iKIR was significantly associated with improved overall survival. These findings have not been previously validated in pediatric studies and may have clinical relevance for haploidentical transplantation population, pending confirmation in larger cohorts.

## Introduction

Allogeneic hematopoietic stem cell transplantation (HSCT) represents a curative therapy for pediatric malignancies and non-malignant disorders. Recent advances in conditioning regimens and optimization of supportive care have significantly reduced non-relapse morbidity and mortality, particularly in the context of unrelated donor transplantation (1). Consequently, the prognosis associated with unrelated donor transplantation has become comparable to that of transplantation from HLA-identical sibling donors (2).

In haploidentical transplantation, the availability of multiple potential donors nearly necessitates the best donor selection, which remains a critical consideration (3). Nevertheless, relapse continues to be a predominant contributor to therapeutic failure in haploidentical HSCT, despite notable clinical improvements, underscoring the urgent need for innovative strategies to refine donor selection protocols and improve long-term outcomes. In this regard, the role of natural killer (NK) cell alloreactivity mediated by killer immunoglobulin-like receptors (KIRs) in modulating immune reconstitution and relapse after transplantation has garnered considerable attention, although its clinical implications remain incompletely defined.

KIRs, which are expressed on NK cells, regulate immune responses through interactions with human leukocyte antigen (HLA) ligands. These receptors are classified as activating KIRs (aKIRs) and inhibitory KIRs (iKIRs), with the latter recognizing HLA-C and HLA-Bw4 ligands, while the former enhance cytotoxic activity. Emerging evidence suggests that KIR diversity may influence relapse and survival through various alloreactive mechanisms, including licensing, missing-ligand recognition, and haplotype diversity. For instance, in haploidentical HSCT utilizing post-transplant cyclophosphamide (PTCy), mismatches in iKIR or the presence of KIR haplotype B donors have been associated with improved survival and reduced relapse rates in myeloid malignancies (4, 5). Conversely, aKIRs have been correlated with increased rates of graft-versus-host disease, relapse, and mortality in mixed cohorts (6). In matched unrelated transplantation, centromeric B motifs of donor KIRs have been linked to increased non-relapse mortality, emphasizing the necessity for context-specific considerations (7).

In the context of haploidentical HSCT, the European Society for Blood and Marrow Transplantation (EBMT) issued recommendations for haploidentical donor selection in 2019 (2); however, these guidelines do not specify which predictive strategies should be used to assess NK cell alloreactivity. On T cell–depleted platforms, the designation of an NK alloreactive donor is prioritized as the second criterion of interest, whereas on T cell–replete platforms, the criterion of a donor with a KIR–ligand match for the recipient is ranked seventh of eight, likely due to the overshadowing effect of T-lymphocyte alloreactivity on NK cell responses.

Advancements in sequencing technology have facilitated high-resolution genotyping of KIR genes at the allelic level, revealing that simple presence/absence typing is inadequate for determining the functional status of KIRs. For example, *KIR3DL1* exhibits variability in expression patterns; specifically, KIR3DL1*004—the third most prevalent *KIR3DL1* allele, representing 17% of all *KIR3DL1* genes—is not expressed on the cell surface. Therefore, relying solely on genetic presence versus absence may lead to erroneous conclusions regarding functional capabilities (8).

It is important to note that most donor KIR genotype–based prediction models have not been successfully validated to date (8–10). Notably, there is a scarcity of studies focusing on this topic in pediatric recipients. In children with acute lymphoblastic leukemia (ALL) undergoing myeloablative HSCT, donor KIR B haplotypes and the centromeric–telomeric (*ct*)-*KIR* score have been associated with a lower incidence of relapse and prolonged event-free survival, regardless of minimal residual disease status (1). This finding highlights the potential utility of *ct-KIR* scoring as a donor selection tool, particularly when multiple HLA-matched donors are available.

A recent comprehensive analysis of pediatric acute leukemia patients who underwent unrelated donor transplantation revealed that KIR–ligand mismatch, KIR gene content, KIR2DS1 mismatching, and centromeric/telomeric haplotypes did not show clear correlations with relapse or disease-free survival (11). These discrepancies may reflect differences in transplantation protocols, disease subtypes, and patient demographics (1, 8). In the present study, we examined the impact of NK cell alloreactivity, as predicted by KIR–ligand interactions, on clinical outcomes in a Chinese pediatric cohort—a population often underrepresented in the international literature. We also used high-resolution KIR genotyping data from donors and recipients to assess the clinical significance of allele mismatches. Our findings indicate that alloreactivity predicted by the interaction between donor KIR3DL2 and its cognate ligands HLA-A3/A11 significantly reduced relapse risk—a factor rarely addressed in previous studies.

## Materials and methods

### Study design and patient population

This retrospective cohort study was conducted in pediatric patients undergoing their first haploidentical transplantation following the Beijing protocol at our hospitals from 2013 to 2023. The study included children diagnosed with acute myeloid leukemia (AML), acute lymphoblastic leukemia (ALL), or other malignant hematological diseases. All patients received modified myeloablative conditioning regimens that included total body irradiation, busulfan, or fludarabine, depending on the patient’s comorbidities.

Graft-versus-host disease (GVHD) prophylaxis consisted of anti-thymoglobulin (ATG), cyclosporin A (CsA), methotrexate (MTX), and mycophenolate mofetil (MMF). Patients were excluded if they had previously undergone solid organ transplantation, had incomplete KIR genotyping data, or lacked samples for retesting. The study was approved by the ethics committee (No. S2025-014-01), and informed consent was obtained in accordance with the Declaration of Helsinki.

### KIR and HLA genotyping

All donors and patients were tested for KIR genes. The presence or absence of various KIR genes (*KIR2DL1*, *KIR2DL2/3*, *KIR2DL4*, *KIR2DL5A*, *KIR2DL5B*, *KIR2DS1*, *KIR2DS2*, KIR2DS3, *KIR2DS4*, *KIR2DS5*, *KIR3DL1/3DS1*, *KIR3DL2*, *KIR3DL3*) and two KIR pseudogenes (*KIR2DP1* and *KIR3DP1*) was determined using a commercially available KIR-SSO typing kit (Immucor Transplant Diagnostics, USA). Amplicons were quantified with the Luminex LABScanTM 100 flow analyzer. Allelic subtypes for *KIR2DS4* were resolved using probe-based PCR (probes 145, 175, and 234).

Three inhibitory KIR genes—*KIR2DL1*, *KIR3DL1*, and *KIR2DL3*—were prioritized for high-resolution genotyping using next-generation sequencing (NGS) to identify allelic variants (GenDx, Netherlands) in both donors and patients. Class I and II HLA loci were typed for all recipients and donors using sequencing-based typing (SBT) with GenDx excellerator kits (GenDX, Netherlands). Epitope ligand groups (HLA-C1/C2, -Bw4/Bw6, -A3/A11) were assigned, and HLA-B alleles were grouped into Bw4-80I/Bw-80T/Bw6 epitope-bearing ligands based on information retrieved from the database (https://www.ebi.ac.uk/ipd/kir/ligand.html) and relevant publications (12, 13). HLA typing results for patients and donors were also used to calculate subtype frequencies with the R package midasHLA version 1.14.0 (14).

### KIR modeling and alloreactivity prediction

Various models have been developed to explain how KIRs and their cognate ligands influence clinical outcomes (2, 3). The ligand–ligand model compares the KIR ligands of the donor and recipient without considering KIR genotyping; donor NK cells will be alloreactive toward host cells if the recipient lacks HLA class I molecules that are present in the donor. The receptor–ligand (or missing-ligand) model takes into account both donor KIR and its HLA ligand in the recipient: if at least one KIR does not recognize its cognate HLA molecules, NK cell inhibition in the donor is reduced, leading to increased cytotoxic activity. This model does not consider the education of donor NK cells.

The educational model encompasses both donor and recipient HLA class I molecules, as well as donor KIR typing. If the donor carries both a KIR and its ligand, the donor NK cells will be licensed and exhibit full alloreactivity. Conversely, if the donor has the KIR but lacks its ligand, the NK cells will remain unlicensed.

The haplotype-based model evaluates the composition of activating and inhibitory genes within KIR haplotypes (2, 19), which are split into two parts: centromeric (*Cen*, A/B motif) and telomeric (*Tel*, A/B motif). *KIR* A/B haplotypes can be distinguished by their characteristic gene content. The A haplotype comprises seven genes (*KIR3DL3*, *KIR2DL3*, *KIR2DL1*, *KIR2DL4*, *KIR3DL1*, *KIR2DS4*, and *KIR3DL2*) and two pseudogenes (*KIR2DP1* and *KIR3DP1*), while the B haplotype is characterized by the presence of at least one of eight genes (*KIR2DS2*, *KIR2DL2*, *KIR2DL5B*, *KIR2DS3*, *KIR3DS1*, *KIR2DL5A*, *KIR2DS5*, and *KIR2DS1*) (2).

To elucidate the combined effects of KIRs, scoring systems have been developed to integrate data on KIRs and their respective ligands. The B content score, which ranges from 0 to 4, is derived by counting the number of B motifs. The *ct-KIR* score is assigned based on the presence of CenB and TelB motifs: a score of 0 for donors with CenB– and TelB+, a score of 1 for those with either CenB+ and TelB+ or CenB– and TelB–, and a score of 2 for those with CenB+ and TelB–. The KIR matching model quantifies the presence of aKIR and/or iKIR genes in the donor that are absent in the recipient, and vice versa.

These models have primarily focused on the presence or absence of KIR genes, neglecting the implications of allelic polymorphism, which can result in KIR molecules with significant biological differences.

The presence of NK cell alloreactivity was initially assessed using the educational model, which is predominantly applied to interactions between the four main iKIR genes and their HLA ligands in the recipient: 2DL1–C2, 2DL2/3–C1, 3DL1–Bw4, and 3DL2–A3/A11. An additional educational pathway involves the activating KIR2DS1 and its cognate C2 ligand (15). Donors with the 2DS1+/C1+ genotype produce NK cells that are fully educated and capable of recognizing their ligands on C2+ recipient cells. In contrast, donors with the 2DS1+/C1– genotype generate hyporesponsive NK cells, regardless of the ligands expressed by leukemic cells (16).

In high-resolution typing, *KIR3DL1* allotypes were classified as high expression (KIR3DL1*001, KIR3DL1*015015), low expression (KIR3DL1*005, KIR3DL1*007), or null/lack-of-expression (KIR3DL1*004) (17). The dimorphism between isoleucine and threonine at position 80 of HLA-Bw4 was categorized as high (Bw4-80I) or low (Bw4-80T) expression. Further comparisons were conducted to assess the inhibitory effects of various allelic combinations (80I/T).*KIR2DL1* allotypes were categorized as KIR2DL1*002/001g, KIR2DL1*003, KIR2DL1*004g, and null allele group (KIR2DL1*0320102N). Notably, alleles with arginine at amino acid position 245 (R^245^) show lower expression and weaker inhibitory signaling than those with cysteine at the same position (C^245^) (18). *KIR3DL1*, *KIR2DL1*, and *KIR2DL3* were present in nearly all donors and recipients, making receptor–receptor mismatch analysis based on gene presence or absence at the allelic level unfeasible.

Secondly, models integrating information on varying degrees of iKIR-mediated inhibition and KIR2DS1 activation were evaluated for their ability to predict relapse risk and overall survival, following the methodology of Dhuyser (3). The qualitative value of each individual KIRxDLx education model (Education–2DL1, –2DL2/3, –3DL1, –3DL2) was first assessed. This was followed by a quantitative analysis in which the number of iKIR models predicting alloreactivity was counted, yielding an integer value from 0 to 4. Because of the limited sample size, statistical power was insufficient for subgroup analysis based on these values. Therefore, patients without predicted alloreactivity were compared with those showing predictions from 1 to 4 models (indicating at least one model predicted alloreactivity).

The study also validated the association between haplotype motif–based scores and clinical outcomes (19, 20). Given the interaction between donor KIRs and recipient ligands, KIR functionality was contingent on the presence of cognate ligands expressed by the recipient’s HLA molecules. The count functional inhibitory KIR score (CF-iKIR score) was calculated as follows: CF-iKIR score = (1 if functional KIR2DL1) + (1 if functional KIR2DL2 and/or functional KIR2DL3) + (1 if functional KIR3DL1) (21). The weighted inhibitory score was calculated as: inhibitory score = (1 if functional KIR2DL1) + (1 if strong functional KIR2DL2 or 0.5 if weak functional KIR2DL2) + (0.75 if functional KIR2DL3) + (1 if functional KIR3DL1) (8, 21).

Additionally, the *ct-KIR* score was tested (1), as it has been identified as a relapse risk factor in childhood acute lymphoblastic leukemia. The KIR B content score was calculated using an online calculator (https://www.ebi.ac.uk/ipd/kir/matching/b_content/). All individuals were categorized as homozygous A haplotype (A/A) or carrying at least one group B haplotype (B/x). Bx haplotypes with a prevalence exceeding 20 donors in the cohort were assessed and labeled according to the Allele Frequency Net Database (http://www.allelefrequencies.net/kir6001a.asp) for further study (22, 23).

### Clinical analysis

The primary endpoints were relapse incidence and overall survival (OS). OS was defined as the time from transplantation to death from any cause, with censoring at the last follow-up for patients who were alive. Kaplan–Meier curves were used to compare survival rates across KIR-defined subgroups. The cumulative incidence of relapse (CIR) was estimated using Fine and Gray’s method, with NRM treated as a competing risk. Unadjusted outcomes were compared between groups with or without predicted alloreactivity using an indicator variable for transplants. Multivariate analysis was performed using Cox proportional hazards regression models, adjusting for clinical covariates such as donor age, donor sex (female), patient disease type, and HLA matching within donor/recipient pairs. Each KIR-based variable was individually examined by incorporating each into the multivariate model. Cases with incomplete data were excluded from the analysis.

Descriptive statistics were used to summarize population characteristics. Continuous variables are referred to as medians, and categorical variables as counts (%). Direct counting based on the presence or absence of each KIR gene was used to determine observed gene frequencies in the studied population. All statistical tests were two-sided, and p values less than 0.05 were considered statistically significant. Data were collected up to December 31, 2023. Statistical analysis was performed using the graphical user interface for R, EZR version 1.32 (24).

## Results

### Population characteristics and NK cell alloreactivity prediction

This analysis included 189 patients with a median age of 6.0 years (range, 1–16 years). Demographic and transplant-related characteristics are summarized in **Table 1**. The initial diagnoses were acute myeloid leukemia/myelodysplastic syndrome in 96 patients (50.8%), acute lymphoblastic leukemia in 74 patients (39.2%), juvenile myelomonocytic leukemia (JMML) in 10 patients, mixed phenotype acute leukemia in 5 patients, and other diseases in 4 patients (including 1 case of acute non-lymphocytic leukemia, 1 case of T-lymphoblastic lymphoma, and 2 cases of myeloid sarcoma).

**Table 1:** Population characteristics.

Variable		No. (n = 189)
Recipient’s age (year)	Median (range)	4 (1–16)
Disease diagnosis (%)	AML/MDS	96 (50.8%)
	ALL	74 (39.2%)
	JMML	10 (5.3%)
	MPAL	5 (2.6%)
	Others*	4 (2.1%)
Donor relationship	Parent	168 (88.9%)
	Sibling	21 (11.1%)
ABO incompatibility	Match	100 (52.9%)
	Major mismatch	40 (21.2%)
	Minor mismatch	34 (18.0%)
	Bidirectional mismatch	15 (7.9%)
HLA match	Mismatch	178 (94.2%)
	Full match	11 (5.8%)
TBI	yes	43 (22.8%)
	no	146 (77.2%)
Disease status at transplant	CR1	131 (69.3%)
	CR2	30 (15.9%)
	NR	28 (14.8%)
Donor Sex	Female	138 (73.0%)
	Male	51 (27.0%)
Donor centromeric motif	AA	98 (51.9%)
	Bx	91 (48.1%)
Donor telomeric motif	AA	111 (58.7%)
	Bx	78 (41.3%)
CF-iKIR score	Median (range)	1.5 (1-3)
Inhibitory KIR score	Median (range)	1.75 (0.75-4.75)
B content score	Neutral	133 (70.4%)
	Better/Best	56 (29.6%)
Relapse days	Median (range)	208 (38-1446)

*Others included: ANLL, acute non-lymphocytic leukemia; LBL, lymphoblastic lymphoma; MS, myeloid sarcoma. ALL, acute lymphocytic leukemia; AML, acute myelocytic leukemia; MDS, myelodysplastic syndromes; JMML, juvenile myelomonocytic leukemia; MPAL, mixed-phenotype acute leukemia; CF-iKIR score, count of functional inhibitory KIR score; TBI, total body irradiation; CR, complete remission; NR, non-remission.

There were 47 relapses, with a median time to relapse of 84 days (range, 38–1,446 days), and 26 deaths, including 4 cases of non-relapse mortality (NRM). The median follow-up duration was 812 days (range, 85–3,193 days). The inhibitory KIR score and CF-iKIR score, derived from the presence of donor inhibitory KIRs and cognate HLA ligands in the recipient, had median values of 1.5 (range, 1–3) for the CF-iKIR score and 1.75 (range, 0.75–4.75) for the inhibitory KIR score.

The number of patients with positive predicted alloreactivity based on the four educational iKIR models ranged from 12 to 37. Collectively, 86 patients demonstrated predicted cytotoxicity with at least one positive iKIR alloreactivity, referred to as Synthesis-iKIR. In the activating KIR2DS1–education model, 36 patients exhibited predicted alloreactivity. In total, 107 patients (56.6% of the cohort) had positive predicted alloreactivity. These results are summarized in **Table 2**.

**Table 2:** Impact of NK cell predicted alloreactivity on 5-year relapse incidence and overall survival.

Variable		Relapse (95% CI)	Gray’s test	OS (95%)	log-rank test
KIR3DL1	Non-ALLO (n = 150)	0.286 (0.201-0.376)	0.558	0.814 (0.723-0.877)	0.336
	ALLO (n = 37)	0.330 (0.149-0.524)		0.912 (0.752-0.971)	
	Strong inhib. (n = 94)	0.208 (0.128-0.303)	0.061	0.843 (0.733-0.910)	0.759
	Weak/noninhib. (n = 83)	0.373 (0.241-0.506)		0.823 (0.681-0.906)	
KIR2DL1	Non-ALLO (n = 159)	0.316 (0.229-0.407)	0.198	0.801 (0.711-0.865)	0.027
	ALLO (n = 27)	0.175 (0.049-0.365)		1.000	
KIR2DL2/3	Non-ALLO (n = 174)	0.277 (0.201-0.357)	0.643	0.844 (0.771-0.896)	0.906
	ALLO (n = 12)	0.452 (0.095-0.765)		0.700 (0.225-0.918)	
KIR3DL2	Non-ALLO (n = 164)	0.318 (0.236-0.403)	0.012	0.804 (0.715-0.868)	0.125
	ALLO (n = 23)	0.091 (0.004-0.347)		1.000	
KIR2DS1	Non-ALLO (n = 151)	0.274 (0.190-0.365)	0.495	0.830 (0.746-0.889)	0.525
	ALLO (n = 36)	0.352 (0.179-0.530)		0.851 (0.620-0.943)	
Synthesis-iKIR	Non-Allo (n = 101)	0.301 (0.212-0.396)	0.126	0.763 (0.650-0.844)	0.008
	ALLO (n = 86)	0.277 (0.158-0.410)		0.912 (0.784-0.965)	
Combined iKIR/2DS1	Non-ALLO (n = 80)	0.307 (0.204-0.415)	0.148	0.729 (0.586-0.830)	0.004
	ALLO (n = 107)	0.268 (0.171-0.375)		0.902 (0.806-0.952)	
Inhibitory score	<=median (n = 93)	0.280 (0.168-0.402)	0.983	0.843 (0.740-0.908)	0.526
	> median (n = 94)	0.304 (0.198-0.416)		0.825 (0.704-0.900)	
*ct-KIR* score	1 (n = 176)	0.298 (0.219-0.382)	0.975	0.826 (0.746-0.884)	0.571
	2 (n = 13)	na		na	
CF-iKIR score	<=1.5 (n = 138)	0.336 (0.234-0.317)	0.182	0.819 (0.719-0.886)	0.553
	>1.5 (n = 50)	0.192 (0.093-0.317)		0.857 (0.701-0.935)	
Centromic haplotype	Bx (n = 91)	0.318 (0.196-0.447)	0.923	0.801 (0.688-0.877)	0.270
	AA (n = 98)	0.276 (0.182-0.378)		0.867 (0.745-0.933)	
Telomeric haplotype	Bx (n = 78)	0.321 (0.193-0.457)	0.911	0.863 (0.731-0.933)	0.405
	AA (n = 111)	0.271 (0.184-0.366)		0.814 (0.710-0.883)	
B content	neutral (n = 133)	0.270 (0.186-0.362)	0.558	0.836 (0.747-0.896)	0.975
	better/best (n = 56)	0.344 (0.194-0.499)		0.832 (0.662-0.921)	
Genotype	G2 (n = 28)	0.384 (0.117-0.653)	0.874	0.855 (0.650-0.991)	0.656
	G8 (n = 17)	0.176 (0.041-0.390)		0.941 (0.650-0.991)	
	other Bx (n = 45)	0.331 (0.173-0.497)		0.855 (0.477-0.967)	
	AA (n = 99)	0.274 (0.180-0.375)		0.802 (0.690-0.878)	

CF-iKIR, count of the functional inhibitory KIR; *ct-KIR*, centromic/telomeric KIR score.

### Allotype frequency distribution and allelic polymorphism for donor and recipient

**Figure 1:**
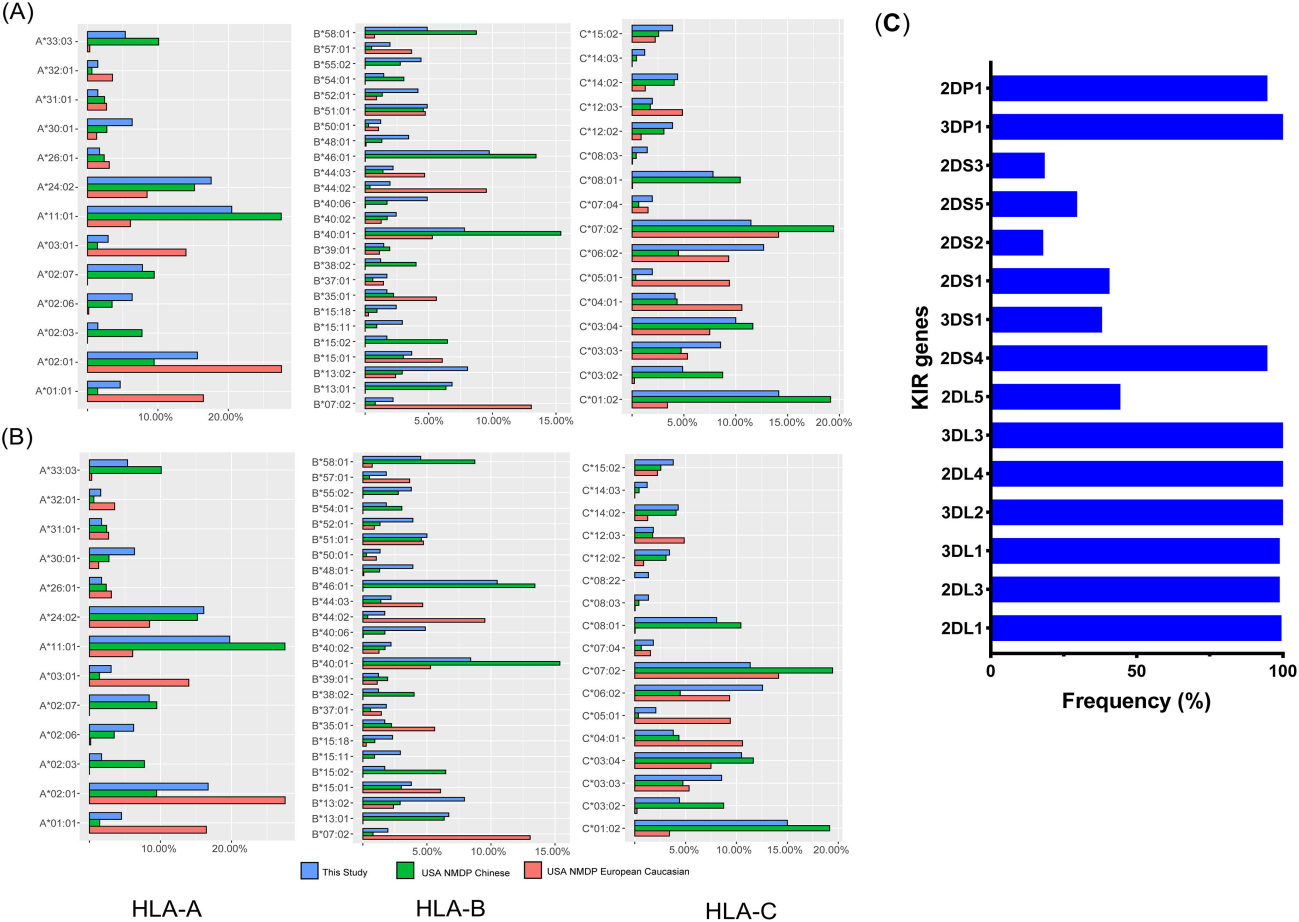
Frequencies of HLA and KIR genes. Frequencies of common HLA subtypes in donors **(A)** and recipients **(B)**, compared with those in Caucasian and Chinese populations retrieved from the NMDP database (National Marrow Donor Program); **(C)** Frequencies of KIR genes in donors.

Four inhibitory genes (*KIR2DL1*, *KIR2DL3*, *KIR3DL1*, and *KIR3DL2*) were present in nearly all donors, while the activating KIR genes *KIR2DS4*, *KIR3DS1*, and *KIR2DS1* were found in 94.7%, 38.1%, and 40.7% of donors, respectively (**Figure 1C**). HLA genotyping within our cohort was counted and compared across different populations, as illustrated in **Figures 1A** and **B**. Consistent with previous reports, the allotype distributions of HLA-A, -B, and -C loci in the Chinese population differed from those in European Caucasians, despite our limited sample size.

Among the Chinese cohort, the most frequently observed subtypes were A*11:01, B*40:01, and C*01:02, whereas A*01:01, B*07:02, and C*07:02 were predominant among European Caucasians. The frequency of A*01:01, A*03:01, B*07:02, B*44:02, C*05:01, and C*04:01 among the Caucasian population was significantly higher than in the Chinese cohort. In contrast, A*33:03, A*02:07, B*46:01, B*13:01, C*08:01, and C*01:02 were more common in the Chinese cohort. According to epitope statistics, 77 patients had HLA-A3/A11, 118 had Bw4, and 174 were C1+.

In the context of allelic distribution, KIR2DL1*003 was the predominant allele among donors, exhibiting a positivity rate of 81.0%, followed by KIR2DL1*002 (13.7%), KIR2DL1*004 (2.5%), and KIR2DL1*069 (2.2%). For the *KIR3DL1* locus, KIR3DL1*015 was the most prevalent allele, with a positivity rate of 63.7%, followed by KIR3DL1*005 and KIR3DL1*007, which had positivity rates of 14.8% and 9.9%, respectively. The allele distribution among recipients was generally similar to that of donors, except for three novel low-frequency alleles—KIR3DL1*038, KIR3DL1*077, and KIR2DL3*006—present in recipients at frequencies of 0.6%, 0.3%, and 0.6%, respectively. Allele frequencies for the three common KIR genes in donors and recipients are summarized in **Table 3**.

**Table 3:** Allele frequencies (%) of the KIR3DL1, KIR2DL1, and KIR2DL3 genes in donors and recipients.

3DL1	Expression	Donor	Recipient	2DL3	Donor	Recipient	2DL1	Residues at 245	Donor	Recipient
*001	high	7.8	7.0	*001	81.5	81.0	*002	R	13.7	14.6
*005	low	14.8	11.3	*002	13.6	13.2	*003	R	81.0	78.4
*007	low	9.9	8.5	*015	2.0	1.4	*004	C	2.5	2.8
*008	high	0.3	0.6	*019	0.9	1.1	*007	C	0.3	0.3
*015	high	63.7	68.3	*022	0.6	0.9	*034	R	0.3	0.3
*020	high	1.7	2.1	*023	1.1	1.4	*069	R	2.2	3.7
*029	high	1.5	1.2	*026	0.6	0.3				
*002	high	0.3	0	*030	0.3	0				
*038	high	0	0.6	*006	0	0.6				
*077	unknown	0	0.3							

### Effect of predictive NK alloreactivity on relapse and overall survival by individual or combinatorial interactions

We tested potential alloreactivity through the interaction of individual KIRs with HLA ligands within an educational model framework. NK cell alloreactivity was classified as ALLO (indicating the presence of predicted alloreactivity) or Non-ALLO (indicating the absence of predicted alloreactivity). Unlicensed NK cells, which typically exhibit hyporesponsiveness to stimuli, were classified as Non-ALLO and compared with NK cells showing predicted alloreactivity.

**Figure 2:**
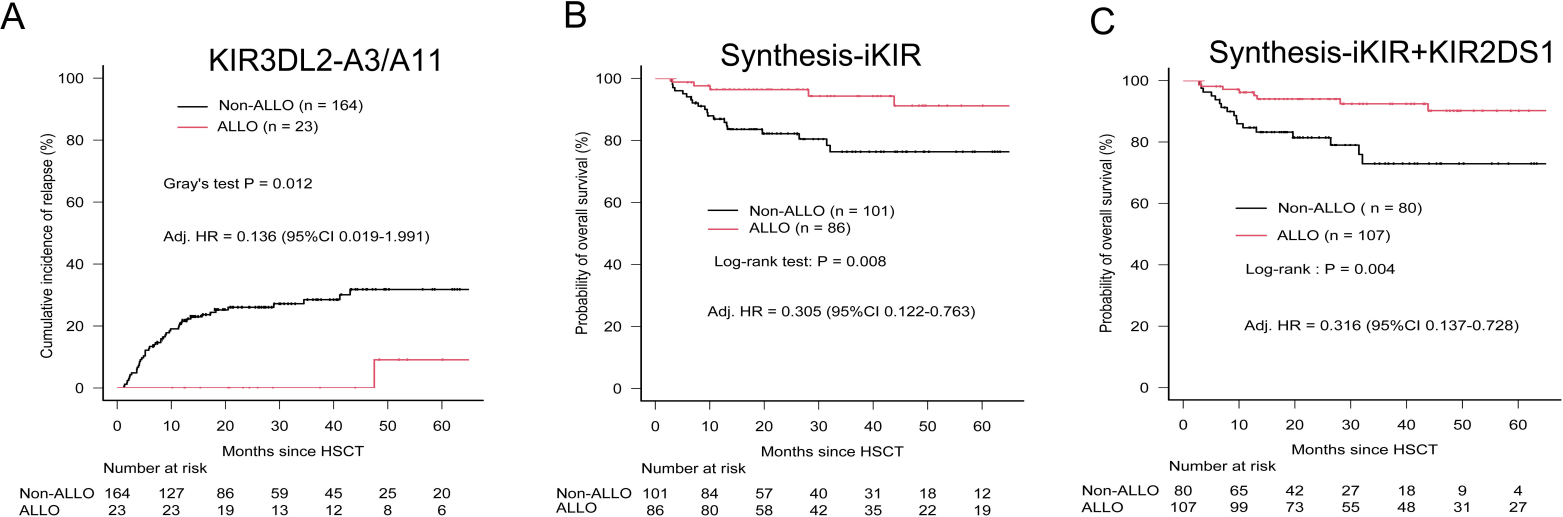
Relapse incidence and overall survival according to predicted alloreactivity. **(A)** Cumulative incidence of relapses according to the presence or absence of alloreactivity predicted by the KIR3DL2-licensed model. **(B)** OS stratified by predicted alloreactivity from the Synthesis-iKIR model, in which at least one of four iKIR–ligand interactions predicted alloreactivity (ALLO). **(C)** OS stratified by predicted alloreactivity from the Synthesis-iKIR plus KIR2DS1 model. Any interaction predicting alloreactivity was defined as presence (ALLO). P-values were calculated using Gray’s test for CIR and the log-rank test for OS. Adjusted HRs (hazard ratios) were calculated in multivariate Cox regression models.

With the exception of the KIR3DL2–A3/A11 combination, patients whose donors exhibited non-inhibiting KIR–ligand interactions or those with activating KIR2DS1–ligand interactions did not show a lower risk of relapse, challenging their utility as prognostic indicators in pediatric haploidentical transplantation. The 5-year cumulative incidence of relapse (CIR) for patients classified as Non-ALLO based on KIR3DL2–A3/A11 interactions was significantly higher (0.318; 95% CI, 0.236–0.403) compared with those classified as ALLO (0.091; 95% CI, 0.004–0.347) (p = 0.012) (**Table 2**). The influence of the KIR3DL2–A3/A11 combination on relapse risk remained significant after adjusting for clinical factors, with an 86.4% reduction in relapse risk for patients with negative A3/A11 receiving both KIR3DL2+ and A3/A11+ grafts (adjusted HR, 0.136; p = 0.0489) (**Table 4**, **Figure 2**).

**Table 4:** Cox multivariate analysis of the effect of NK cell-predicted alloreactivity on relapse and survival.

Variable		Adj. HR	P
Relapse			
KIR3DL2 educational model	ALLO *vs*. Non-ALLO	0.136 (0.019-0.991)	4.89×10^-2^
Disease status at transplant	NR *vs*. CR	3.351 (1.756-6.396)	2.0×10^-4^
Overall survival			
Synthesis-iKIR	ALLO *vs*. Non-ALLO	0.305 (0.122-0.763)	1.06×10^-2^
Disease status at transplant	NR *vs*. CR	3.126 (1.305-7.490)	1.11×10^-2^
Overall survival*			
Combined iKIR/KIR2DS1	ALLO *vs*. Non-ALLO	0.316 (0.137-0.728)	6.79×10^-3^
Disease status at transplant	NR *vs*. CR	2.917 (1.218-6.986)	1.63×10^-2^

*Considering both Synthesis-iKIR and KIR2DS1 model simultaneously. HR, hazard ratio.

In terms of KIR2DS1–C2 educational interactions, patients exhibiting activating KIR–HLA interactions had a higher 5-year CIR (0.352; 95% CI, 0.179–0.530; n = 36) compared with those without predicted alloreactivity (0.274; 95% CI, 0.190–0.365; n = 151), although this difference did not reach statistical significance (p = 0.495) (**Table 2**).

**Figure 3:**
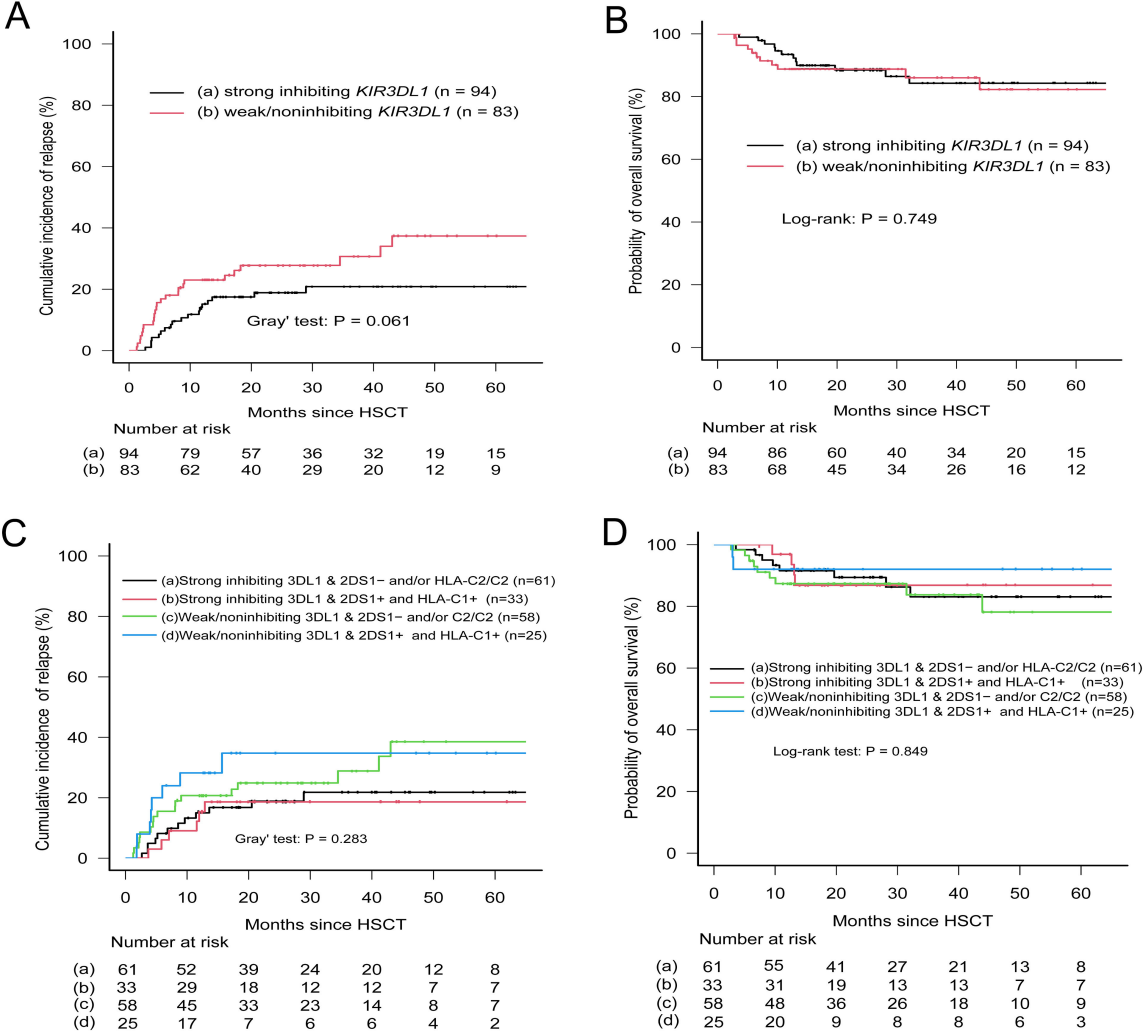
Relapse incidence and overall survival according to donor KIR3DL1 allele expression and patient HLA-Bw4 subtype. **(A, B)** Patients were stratified into strong-inhibiting and weak/non-inhibiting groups based on KIR3DL1/HLA-Bw4 combinations. **(C, D)** Patients were grouped by donor KIR2DS1 status (activating vs. non-activating) and KIR3DL1/HLA-Bw4 subtype. Classification followed Boudreau et al. (32) and Schetelig et al. (9). Groups a and c in panels **(C, D)** included (1) absence of donor KIR2DS1, and (2) presence of donor KIR2DS1 with ligand C2-homozygous patients.

Given that nearly all donors possessed the KIR2DL1-R^245^ type, a grouping analysis was performed for patients based on the presence or absence of allele mismatches. Among patients with the C2+ antigen, the presence of KIR2DL1 allele mismatches within donor–recipient pairs was strongly associated with an elevated risk of relapse (CIR, 0.482; 95% CI, 0.223–0.702; p = 0.023). Conversely, the absence of such mismatches correlated with a lower relapse risk (CIR, 0.296; 95% CI, 0.206–0.393), although no statistically significant difference in CIR was observed across the entire cohort (**Table 5**). Furthermore, KIR2DL1 allele mismatches did not appear to influence OS (**Table 5**). Patients were subsequently grouped according to donor KIR2DS1 status (activating versus nonactivating) and combinations of KIR3DL1–Bw4 (strong inhibiting versus weak/noninhibiting). The CIR curves for the four groups were almost superimposable (**Figure 3**), and further analysis indicated no statistically significant differences in OS among these groups.

**Table 5:** Impact of mismatches within donor/recipient pairs on 5-year relapse incidence and overall survival.

Variable		Relapse (95% CI)	P	OS (95% CI)	P
KIR3DL1					
All patients	Allele mismatch (n = 82)	0.305 (0.185-0.435)	0.922	0.880 (0.782-0.936)	0.363
	Allele match (n = 72)	0.303 (0.193-0.421)		0.802 (0.670-0.885)	
Bw4+ patients	Allele mismatch (n = 50)	0.228 (0.110-0.371)	0.995	0.871 (0.755-0.945)	0.450
	Allele match (n = 42)	0.224 (0.109-0.364)		0.777 (0.600-0.900)	
KIR2DL1					
All patients	mismatch (n = 41)	0.357 (0.188-0.530)	0.348	0.874 (0.722-0.946)	0.921
	match (n = 128)	0.296 (0.206-0.393)		0.822 (0.719-0.890)	
C2+ patients	mismatch (n = 17)	0.482 (0.223-0.702)	0.023	0.801 (0.499-0.932)	0.378
	match (n = 52)	0.275 (0.138-0.430)		0.822 (0.632-0.920)	
KIR2DL3					
All patients	mismatch (n = 45)	0.278 (0.153-0.418)	0.791	0.893 (0.731-0.960)	0.321
	match (n = 120)	0.299 (0.201-0.403)		0.813 (0.705-0.885)	
C1+ patients	mismatch (n = 42)	0.253 (0.134-0.391)	0.944	0.885 (0.712-0.957)	0.427
	match (n =109)	0.266 (0.179-0.362)		0.840 (0.743-0.903)	

We then assessed the influence of KIR haplotypes on relapse incidence and survival. Previous studies have indicated that patients receiving Bx grafts have a lower risk of relapse compared with those with AA donors. However, our analysis did not reveal a significant trend toward a lower CIR for patients with Tel- or Cen-Bx grafts compared with Cen- or Tel-AA donors (p > 0.05) (**Table 2**). Due to the limited number of individuals classified as the best cases, we combined the better and best individuals into a preferred group for comparison with a neutral group. This comparison yielded no significant differences in OS or CIR between the two groups. Similarly, Kaplan–Meier survival curves showed no significant differences in OS among patient groups categorized by donor Cen/Tel haplotypes (p > 0.05; data not shown).

An additional widely used algorithm for donor selection in HSCT is the KIR B content score, which classifies donors as neutral, better, or best, with increasing weighting allocated to donors exhibiting high Cen-B content. The donor cohort in this investigation exhibited a comparable proportion of neutral individuals (70.4%, n = 133) to that reported by Cooley et al. (69.0%), but a higher percentage of better individuals (27.5% *vs.* 20.2%) and a lower proportion of best individuals (1.6% *vs.* 10.8%) (19). Given the limited number of best individuals, we designated the better and best individuals as the preferred group and compared them with the neutral group. This comparison also revealed no statistically significant differences between the two groups with respect to OS and CIR (p > 0.05) (**Table 2**). Furthermore, we completed donor Bx genotype analysis online (AFND, http://www.allelefrequencies.net/kir6001a.asp) to determine potential KIR gene combinations that may affect relapse and survival. Two Bx genotypes with a higher prevalence among donors in the cohort were screened: Genotype 2, characterized by the presence of all KIRs except for KIR2DL2, KIR2DS2, and KIR2DS3, and Genotype 8, which includes all KIRs except for KIR2DL2, KIR2DS2, and KIR2DS5. When compared to other Bx or AA genotypes, regardless of HLA antigen, neither Genotype 8 nor Genotype 2 was associated with CIR or OS across the entire cohort (p > 0.05) (**Table 2**). Utilizing data on KIR haplotypes, we further explored additive models; however, neither the *ct-KIR* nor the weighted inhibitory score exhibited any significant correlation with CIR or OS (**Table 2**).

Finally, multivariate Cox proportional hazards regression analysis demonstrated that the KIR3DL2–A3/A11 combination significantly reduced the incidence of relapse across the whole cohort (adjusted HR, 0.136; p < 0.005) (**Table 4**, **Figure 2**). Synthesis-iKIR, alone or in conjunction with KIR2DS1, had a significant independent effect on OS, with a clinical factor–adjusted HR of 0.305 (95% CI, 0.122–0.763; p < 0.005) and 0.316 (95% CI, 0.137–0.728; p < 0.005), respectively (**Table 4**, **Figure 2**). As anticipated, the disease state at the time of transplantation emerged as an independent prognostic factor (**Table 4**). Other alloreactivity models did not successfully predict relapse or survival among pediatric recipients.

## Discussion

NK cells are believed to play a crucial role in mitigating post-transplant relapse; however, the mechanisms that govern alloreactivity and their clinical significance remain inadequately understood. This study aimed to assess the clinical impact of KIRs and their corresponding ligands on relapse and survival outcomes in a cohort of Chinese pediatric patients undergoing haploidentical transplantation following the Beijing protocol. Our results indicate that the combination of inhibitory KIR3DL2 with A3/A11 was associated with a reduced incidence of relapse, while Synthesis-iKIR was linked to improved survival rates among pediatric patients. Furthermore, the study found that other additive or haplotype motif-based indices did not correlate with clinical outcomes. Although this analysis is limited by its single-center, retrospective design, it represents the first validation of KIR-related effects in Chinese children—a population for whom the Beijing protocol is the standard transplantation regimen.

The prediction of donor NK cell alloreactivity and its clinical outcomes has been investigated primarily in adults undergoing unrelated donor transplantation (3, 25–30), with relatively few studies focusing on pediatric populations (1, 31). Biological differences between adults and children may alter NK allogeneic responsiveness. In children, the presence of a functional thymus has been linked to faster T-cell reconstitution (11), which could reduce NK cell function. Additionally, pediatric patients typically undergo myeloablation, whereas reduced-intensity conditioning is more common in adults. Consequently, it is of great significance to examine the relationship between donor KIR profiles and transplant outcomes within a pure pediatric cohort. The *ct-KIR* score indicated that transplants from donors with a score of 2 were associated with lower relapse rates than those with scores of 1 or 0. Oevermann et al. reported that donors with KIR haplotype B and a high KIR B-content score provided greater protection against relapse in pediatric acute lymphoblastic leukemia (31). In our cohort, patients had *ct-KIR* scores of 1 or 2, but no significant differences in CIR or OS were observed between these groups. Similarly, analyses of KIR haplotypes and B-content scores revealed no significant effects on relapse risk or OS. These negative findings may reflect differences in patient characteristics and transplantation protocols: our study involved Chinese children treated under the Beijing protocol, whereas many prior studies used TCD or PTCy-based approaches. As illustrated in **Figure 1**, the frequencies of HLA subtypes exhibited considerable variation between Chinese and European populations, emphasizing the importance of population- or ethnicity-specific research. For instance, prior studies have indicated that donors homozygous for KIR2DL1-R^245^ confer better survival and reduced relapse risk compared to KIR2DL1-C^245^ donors (33). However, we were unable to thoroughly evaluate the impact of KIR2DL1 dimorphism at position 245 on clinical outcomes due to the skewed distribution in the donor cohort under investigation. Previous research has established that KIR2DL1-C^245^ is rare in East Asian populations (2). Further evidence supporting the influence of population differences is the absence of the KIR3DL1*004 allele in our dataset.

Unexpectedly, we observed that only KIR3DL2, among the four iKIRs, showed an apparent correlation with relapse incidence, although the limited number of ALLO cases may have affected the statistical power (adj. HR 0.136, p < 0.05). *KIR3DL2* is classified as a framework gene and is not included in analyses of KIR haplotypes, B content, CF-iKIR, *ct-KIR*, or inhibitory scores (5). Given our limited dataset, it remains uncertain whether this finding is incidental or genuine; further validation in a larger cohort is necessary to elucidate the clinical importance of KIR3DL2. This study also provides evidence that patients predicted to experience alloreactivity (ALLO) based on the educational iKIR model had a significantly reduced mortality risk (adj. HR 0.305, p < 0.05) (**Table 5**). Moreover, when predicted alloreactivity from the educational KIR2DS1 model was included, the survival benefit remained significant (adj. HR 0.316, p < 0.01). These findings support the hypothesis that a donor’s KIR repertoire benefits the recipient by optimizing signaling through aKIRs while minimizing signaling through iKIRs.

To further substantiate the role of allelic polymorphism, three KIR genes with higher prevalence among donors (*KIR3DL1*, *KIR2DL1*, and *KIR2DL3*) were sequenced using NGS. Research by Boudreau et al. (32) suggested that relapse protection is associated with the inhibitory strength of KIR3DL1/HLA-B combinations. Following their methodology, our cohort was stratified based on the specified criteria, and donor KIR3DL1 allotypes were evaluated in conjunction with either donor or recipient HLA epitopes. Although the strong inhibitory KIR3DL1/Bw4 combination showed a trend toward reduced relapse incidence in univariate analysis (**Table 3**, p = 0.061), this association was not confirmed in the final multivariate model, consistent with recent reports (9, 10, 17). Even when donor KIR2DS1 status was integrated into the final model assessing the inhibitory level of the KIR3DL1/Bw4 combination, clear distinctions between patient groups were not observed (**Figure 3**). Similarly, analysis of the *KIR2DL1* allele indicated that patients with the HLA-C2 epitope who received *KIR2DL1* allele–matched grafts had a reduced relapse risk in univariate analysis (**Table 5**); however, this effect was not evident in the multivariate results. The presence or absence of *KIR2DL3* allele matches between donors and recipients did not influence relapse or OS (**Table 5**). Consequently, while allelic-level analysis may offer new insights into the impact of KIRs on clinical outcomes, its practical significance appears limited. In this context, high-resolution typing yields more detailed information on KIR genes compared with low-resolution typing, but its clinical relevance remains uncertain and requires further validation, as suggested by both our findings and those of previous studies.

This study has certain limitations, including its retrospective design and the relatively small patient cohort. To facilitate clinical application, independent, prospective, large-scale, multi-center studies are warranted. Notably, although adult patients typically exhibit higher relapse rates, the incidence of relapse is lower in pediatric populations, making it difficult for individual centers to assemble a cohort with a sufficient number of positive cases. Consequently, this investigation may have been underpowered, even though most statistical tests yielded non-significant results.

In this study, we established a cohort of pediatric patients diagnosed with malignant hematological tumors and assessed the impact of natural killer (NK) cell alloreactivity on relapse incidence and overall survival within the framework of the Beijing transplantation protocol. We assessed the major categories of KIR-mediated alloreactivity prediction models in our cohort using both KIR presence/absence and allelic typing. Our findings showed that patients with alloreactivity predicted by the KIR3DL2–A3/A11 combination had a significantly lower incidence of relapse. Furthermore, alloreactivity predicted by Synthesis-iKIR—either alone or combined with KIR2DS1 status (Combined iKIR/KIR2DS1)—was associated with improved overall survival. To our knowledge, this is the first study to elucidate the influence of the KIR3DL2–A3/A11 combination on clinical outcomes in pediatric haploidentical HSCT with thymoglobulin. These results underscore the potential of KIR-based NK cell alloreactivity prediction in both clinical research and practice; however, the clinical implications of the KIR3DL2-A3/A11 interaction necessitate further investigation and validation.

## Data Availability

The original contributions presented in the study are included in the article/supplementary material. Further inquiries can be directed to the corresponding authors.

## References

[1] Babor F Peters C Manser AR Glogova E Sauer M Pötschger U. Presence of centromeric but absence of telomeric group B KIR haplotypes in stem cell donors improve leukaemia control after HSCT for childhood ALL. Bone Marrow Transplant. (2019) 54:1847–58. doi: 10.1038/s41409-019-0543-z, PMID: 31089287

[2] Dhuyser A Aarnink A Pérès M Jayaraman J Nemat-Gorgani N Rubio MT. KIR in allogeneic hematopoietic stem cell transplantation: need for a unified paradigm for donor selection. Front Immunol. (2022) 13:821533. doi: 10.3389/fimmu.2022.821533, PMID: 35242134 PMC8886110

[3] Dhuyser A Remen T Pérès M Chamberlain-Evans V Nemat-Gorgani N Campidelli A. Comparison of NK alloreactivity prediction models based on KIR-MHC interactions in haematopoietic stem cell transplantation. Front Immunol. (2023) 14:1028162. doi: 10.3389/fimmu.2023.1028162, PMID: 36936953 PMC10017772

[4] Symons HJ Leffell MS Rossiter ND Zahurak M Jones RJ Fuchs EJ. Improved survival with inhibitory killer immunoglobulin receptor (KIR) gene mismatches and KIR haplotype B donors after nonmyeloablative, HLA-haploidentical bone marrow transplantation. Biol Blood Marrow Transplant. (2010) 16:533–42. doi: 10.1016/j.bbmt.2009.11.022, PMID: 19961944 PMC2848533

[5] Zou J Kongtim P Srour SA Greenbaum U Schetelig J Heidenreich F. Donor selection for KIR alloreactivity is associated with superior survival in haploidentical transplant with PTCy. Front Immunol. (2022) 13:1033871. doi: 10.3389/fimmu.2022.1033871, PMID: 36311784 PMC9606393

[6] Kamenaric MB Jankovic KS Grubic Z Serventi Seiwerth R Maskalan M Nemet D. The impact of KIR2DS4 gene on clinical outcome after hematopoietic stem cell transplantation. Hum Immunol. (2017) 78:95–102. doi: 10.1016/j.humimm.2016.11.010, PMID: 27998801

[7] Bultitude WP Schellekens J Szydlo RM Anthias C Cooley SA Miller JS. Presence of donor-encoded centromeric KIR B content increases the risk of infectious mortality in recipients of myeloablative, T-cell deplete, HLA-matched HCT to treat AML. Bone Marrow Transplant. (2020) 55:1975–84. doi: 10.1038/s41409-020-0858-9, PMID: 32203258 PMC8852820

[8] Schetelig J Baldauf H Koster L Kuxhausen M Heidenreich F de Wreede LC. Haplotype motif-based models for KIR-genotype informed selection of hematopoietic cell donors fail to predict outcome of patients with myelodysplastic syndromes or secondary acute myeloid leukemia. Front Immunol. (2020) 11:584520. doi: 10.3389/fimmu.2020.584520, PMID: 33542712 PMC7851088

[9] Schetelig J Baldauf H Heidenreich F Massalski C Frank S Sauter J. External validation of models for KIR2DS1/KIR3DL1-informed selection of hematopoietic cell donors fails. Blood. (2020) 135:1386–95. doi: 10.1182/blood.2019002887, PMID: 31932846 PMC7162689

[10] Schetelig J Baldauf H Heidenreich F Hoogenboom JD Spellman SR Kulagin A. Donor KIR genotype based outcome prediction after allogeneic stem cell transplantation: no land in sight. Front Immunol. (2024) 15:1350470. doi: 10.3389/fimmu.2024.1350470, PMID: 38629074 PMC11019434

[11] Verneris MR Miller JS Hsu KC Wang T Sees JA Paczesny S. Investigation of donor KIR content and matching in children undergoing hematopoietic cell transplantation for acute leukemia. Blood Adv. (2020) 4:1350–6. doi: 10.1182/bloodadvances.2019001284, PMID: 32267930 PMC7160272

[12] Cooley S Parham P Miller JS. Strategies to activate NK cells to prevent relapse and induce remission following hematopoietic stem cell transplantation. Blood. (2018) 131:1053–62. doi: 10.1182/blood-2017-08-752170, PMID: 29358179 PMC5863700

[13] Pollock NR Harrison GF Norman PJ. Immunogenomics of killer cell immunoglobulin-like receptor (KIR) and HLA Class I: coevolution and consequences for human health. J Allergy Clinic Immunol Pract. (2022) 10:1763–75. doi: 10.1016/j.jaip.2022.04.036, PMID: 35561968 PMC10038757

[14] Migdal M Ruan D Forrest W Horowitz A Hammer C. MiDAS-meaningful immunogenetic data at scale. PloS Comput Biol. (2021) 17:e1009131. doi: 10.1371/journal.pcbi.1009131, PMID: 34228721 PMC8284797

[15] Pende D Marcenaro S Falco M Martini S Bernardo ME Montagna D. Anti-leukemia activity of alloreactive NK cells in KIR ligand-mismatched haploidentical HSCT for pediatric patients: evaluation of the functional role of activating KIR and redefinition of inhibitory KIR specificity. Blood. (2009) 113:3119–29. doi: 10.1182/blood-2008-06-164103, PMID: 18945967

[16] Venstrom JM Pittari G Gooley TA Chewning JH Spellman S Haagenson M. HLA-C-dependent prevention of leukemia relapse by donor activating KIR2DS1. N Engl J Med. (2012) 367:805–16. doi: 10.1056/NEJMoa1200503, PMID: 22931314 PMC3767478

[17] Guethlein LA Beyzaie N Nemat-Gorgani N Wang T Ramesh V Marin WM. Following transplantation for acute myelogenous leukemia, donor KIR cen B02 Better protects against relapse than KIR cen B01. J Immunol. (2021) 206:3064–72. doi: 10.4049/jimmunol.2100119, PMID: 34117109 PMC8664929

[18] Wright PA van de Pasch LAL Dignan FL Kichula KM Pollock NR Norman PJ. Donor KIR2DL1 allelic polymorphism influences posthematopoietic progenitor cell transplantation outcomes in the T cell depleted and reduced intensity conditioning setting. Transplant Cell Ther. (2024) 30:488.e1–.e15. doi: 10.1016/j.jtct.2024.02.014, PMID: 38369017 PMC11056303

[19] Cooley S Weisdorf DJ Guethlein LA Klein JP Wang T Le CT. Donor selection for natural killer cell receptor genes leads to superior survival after unrelated transplantation for acute myelogenous leukemia. Blood. (2010) 116:2411–9. doi: 10.1182/blood-2010-05-283051, PMID: 20581313 PMC2953880

[20] Cooley S Weisdorf DJ Guethlein LA Klein JP Wang T Marsh SG. Donor killer cell Ig-like receptor B haplotypes, recipient HLA-C1, and HLA-C mismatch enhance the clinical benefit of unrelated transplantation for acute myelogenous leukemia. J Immunol. (2014) 192:4592–600. doi: 10.4049/jimmunol.1302517, PMID: 24748496 PMC4031316

[21] Boelen L Debebe B Silveira M Salam A Makinde J Roberts Ch. Inhibitory killer cell immunoglobulin-like receptors strengthen CD8+ T cell. Sci Immunol. (2018) 3:eaao2892. doi: 10.1126/sciimmunol.aao2892, PMID: 30413420 PMC6277004

[22] Fein JA Shouval R Krieger E Spellman SR Wang T Baldauf H. Systematic evaluation of donor-KIR/recipient-HLA interactions in HLA-matched hematopoietic cell transplantation for AML. Blood Adv. (2024) 8:581–90. doi: 10.1182/bloodadvances.2023011622, PMID: 38052043 PMC10837477

[23] González-Galarza FF Takeshita LY Santos EJ Kempson F Maia MH da Silva AL. Allele frequency net 2015 update: new features for HLA epitopes, KIR and disease and HLA adverse drug reaction associations. Nucleic Acids Res. (2015) 43:D784–8. doi: 10.1093/nar/gku1166, PMID: 25414323 PMC4383964

[24] Kanda Y. Investigation of the freely available easy-to-use software ‘EZR’ for medical statistics. Bone Marrow Transplant. (2013) 48:452–8. doi: 10.1038/bmt.2012.244, PMID: 23208313 PMC3590441

[25] Chen DF Prasad VK Broadwater G Reinsmoen NL DeOliveira A Clark A. Differential impact of inhibitory and activating Killer Ig-Like Receptors (KIR) on high-risk patients with myeloid and lymphoid Malignancies undergoing reduced intensity transplantation from haploidentical related donors. Bone Marrow Transplant. (2012) 47:817–23. doi: 10.1038/bmt.2011.181, PMID: 22139069 PMC3629554

[26] Ruggeri L Vago L Eikema D-J de Wreede LC Ciceri F Diaz MA. Natural killer cell alloreactivity in HLA-haploidentical hematopoietic transplantation: a study on behalf of the CTIWP of the EBMT. Bone Marrow Transplant. (2021) 56:1900–7. doi: 10.1038/s41409-021-01259-0, PMID: 33767404

[27] Shimoni A Labopin M Lorentino F Van Lint MT Koc Y Gülbas Z. Killer cell immunoglobulin-like receptor ligand mismatching and outcome after haploidentical transplantation with post-transplant cyclophosphamide. Leukemia. (2019) 33:230–9. doi: 10.1038/s41375-018-0170-5, PMID: 29907809

[28] Willem C Makanga DR Guillaume T Maniangou B Legrand N Gagne K. Impact of KIR/HLA Incompatibilities on NK cell reconstitution and clinical outcome after T cell-replete haploidentical hematopoietic stem cell transplantation with posttransplant cyclophosphamide. J Immunol. (2019) 202:2141–52. doi: 10.4049/jimmunol.1801489, PMID: 30787107

[29] Huang XJ Zhao XY Liu DH Liu KY Xu LP. Deleterious effects of KIR ligand incompatibility on clinical outcomes in haploidentical hematopoietic stem cell transplantation without *in vitro* T-cell depletion. Leukemia. (2007) 21:848–51. doi: 10.1038/sj.leu.2404566, PMID: 17268518

[30] Zhao XY Huang XJ Liu KY Xu LP Liu DH. Prognosis after unmanipulated HLA-haploidentical blood and marrow transplantation is correlated to the numbers of KIR ligands in recipients. Eur J Haematol. (2007) 78:338–46. doi: 10.1111/j.1600-0609.2007.00822.x, PMID: 17378893

[31] Oevermann L Michaelis SU Mezger M Lang P Toporski J Bertaina A. KIR B haplotype donors confer a reduced risk for relapse after haploidentical transplantation in children with ALL. Blood. (2014) 124:2744–7. doi: 10.1182/blood-2014-03-565069, PMID: 25115891 PMC4208288

[32] Boudreau JE Giglio F Gooley TA Stevenson PA Le Luduec JB Shaffer BC. KIR3DL1/HLA-B subtypes govern acute myelogenous leukemia relapse after hematopoietic cell transplantation. J Clin Oncol. (2017) 35:2268–78. doi: 10.1200/jco.2016.70.7059, PMID: 28520526 PMC5501362

[33] Bari R Rujkijyanont P Sullivan E Kang G Turner V Gan K. Effect of donor KIR2DL1 allelic polymorphism on the outcome of pediatric allogeneic hematopoietic stem-cell transplantation. J Clinic Oncol. (2013) 31:3782–90. doi: 10.1200/jco.2012.47.4007, PMID: 24043749 PMC3795888

